# Genomic Characterization of Recrudescent *Plasmodium malariae* after Treatment with Artemether/Lumefantrine

**DOI:** 10.3201/eid2308.161582

**Published:** 2017-08

**Authors:** Gavin G. Rutledge, Ian Marr, G. Khai Lin Huang, Sarah Auburn, Jutta Marfurt, Mandy Sanders, Nicholas J. White, Matthew Berriman, Chris I. Newbold, Nicholas M. Anstey, Thomas D. Otto, Ric N. Price

**Affiliations:** Wellcome Trust Sanger Institute, Hinxton, Cambridge, United Kingdom (G.G. Rutledge, M. Sanders, M. Berriman, C.I. Newbold, T.D. Otto);; Royal Darwin Hospital, Casuarina, Northern Territory, Australia (I. Marr, G.K.L. Huang, N.M. Anstey, R.N. Price);; Menzies School of Health Research and Charles Darwin University, Darwin, Northern Territory, Australia (S. Auburn, J. Marfurt, N.M. Anstey, R.N. Price);; Mahidol University Faculty of Tropical Medicine, Mahidol-Oxford Tropical Medicine Research Unit, Bangkok, Thailand (N.J. White);; University of Oxford Centre for Tropical Medicine and Global Health, Oxford, United Kingdom (N.J. White, R.N. Price);; University of Oxford Weatherall Institute of Molecular Medicine, Oxford (C.I. Newbold)

**Keywords:** recrudescence, Plasmodium malariae, artemether/lumefantrine, malaria, parasites, parasitemia, haplotype, Australia

## Abstract

*Plasmodium malariae* is the only human malaria parasite species with a 72-hour intraerythrocytic cycle and the ability to persist in the host for life. We present a case of a *P. malariae* infection with clinical recrudescence after directly observed administration of artemether/lumefantrine. By using whole-genome sequencing, we show that the initial infection was polyclonal and the recrudescent isolate was a single clone present at low density in the initial infection. Haplotypic analysis of the clones in the initial infection revealed that they were all closely related and were presumably recombinant progeny originating from the same infective mosquito bite. We review possible explanations for the *P. malariae* treatment failure and conclude that a 3-day artemether/lumefantrine regimen is suboptimal for this species because of its long asexual life cycle.

During the past decade, intensification of malaria control efforts has substantially reduced the global burden of malaria from *Plasmodium falciparum*. This trend has often been associated with increased recognition of the burden of malarial disease caused by the other *Plasmodium* species (*1*). *P. malariae,* 1 of the 6 *Plasmodium* species that commonly infect humans, is endemic throughout parts of Africa (*2*,*3*), South America (*4*), Asia, and the western Pacific (*5*). *P. malariae* is unique among the human-infective *Plasmodium* species in having a 72-hour intraerythrocytic lifecycle with variable but often prolonged pre-erythrocytic intrahepatic development (*6*). *P. malariae* can persist in the human host for years and possibly an entire lifetime. Although it is often asymptomatic, chronic parasitemia in endemic areas is associated with substantial rates of illness, including anemia and nephrotic syndrome (*7*–*9*).

A key strategy for malaria elimination is strengthening of health systems to deliver early diagnosis and highly effective therapy. Artemisinin-based combination therapy (ACT) has been central to this approach, with proven efficacy against multidrug-resistant *P. falciparum*, multidrug-resistant *P. vivax,* and *P. knowlesi* (*10*–*13*). In recent years, there have been increasing calls for a universal policy of ACT for all species of malaria (*10*–*13*). However, the efficacy of ACT against *P. malariae* is poorly documented.

Although chronic infection with *P. malariae* is well-recognized (*14*), little is known regarding how the parasites manage to evade host immunity and the intrahost dynamics of the underlying parasite population. Recent advances in molecular genetics have produced the first descriptive analyses of the whole genome sequence of *P. malariae* (*15*,*16*). The *P. malariae* reference genome is 33.6 Mb in size, has 6,540 genes, and has an average guanine plus cytosine content of 24% (*15*).

We report a case of a *P. malariae* infection in a patient residing in a non–malaria-endemic environment that resulted in recrudescence months after treatment with artemether/lumefantrine (AL). By using whole-genome sequencing of isolates from the initial and the recrudescent infections, we show that the 2 major *P. malariae* haplotypes, constituting ≈90% of the parasite load in the initial infection, were cleared successfully by AL, whereas a third haplotype, constituting a minority subpopulation in the initial infection, survived and recrudesced.

## Results

### The Patient

**Figure 1:**
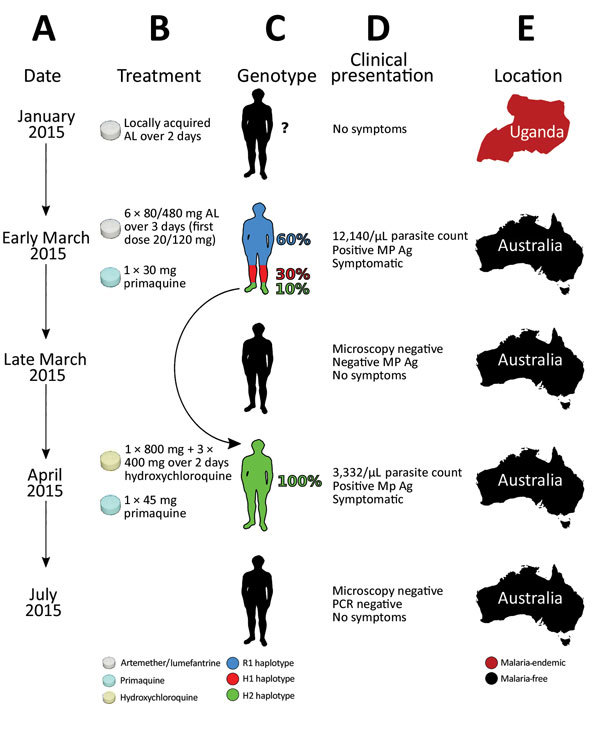
Timeline of the clinical case of a patient with *Plasmodium malariae* infection diagnosed and treated at Royal Darwin Hospital, Darwin, Northern Territory, Australia, March–April 2015, showing the timing (A), treatment (B), parasite’s genotype as inferred from whole-genome sequencing (C), clinical presentation (D), and location (E). The rounded arrow indicates the recrudescence of the minor haplotype 2 in the initial infection to dominate monoclonally in the second infection. AL, artemether/lumefantrine; H1, haplotype 1; H2; haplotype 2; MP Ag, pan-malarial antigen; R1, reference haplotype.

A 31-year-old Uganda-born man, weighing ≈77 kg (≈170 lbs), who had been a resident in Australia for 5 years sought care at Royal Darwin Hospital (Darwin, Northern Territory, Australia) on March 1, 2015, with a 4-day history of fevers and headaches. He had returned to Australia 56 days previously after a 2-week trip to Uganda visiting friends and relatives (Figure 1, panels A, E). He had spent 14 days in a rural malaria-endemic area in eastern Uganda. Although he had not taken regular malaria prophylaxis, he had self-medicated with a locally acquired oral course of AL on the second and third days of his trip, despite being clinically well (Figure 1, panels B, D). He returned to Australia (now a malaria-free country) in January 2015 until seeking care after a short febrile illness in late February. On examination, he had a tympanic temperature of 37.5°C and a heart rate of 110 beats/min but no manifestations of severe malaria. Rapid diagnostic testing with BinaxNOW (Binax, Inc., Inverness Medical Professional Diagnostics, Scarborough, ME, USA) for malaria was positive for aldolase but negative for histidine-rich protein 2. Species-specific PCR was positive for *P. malariae* and negative for all other *Plasmodium* species (Technical Appendix). Thick and thin blood film examination confirmed *P. malariae* parasitemia (12,140 parasites/μL) with all stages of asexual development visible on the blood film (Technical Appendix Figure 1, panels A, B). The blood film was otherwise unremarkable; in particular, no evidence for hyposplenism was found. The patient was not immunosuppressed, and an HIV serologic test was negative. A hepatitis C serologic test was positive but with a viral load that was below the limit of quantification (<12 IU/mL).

The patient was administered a single 20/120 mg tablet of AL on the first day because of a prescribing error but subsequently continued with a supervised standard regimen of 80/480 mg every 12 hours taken with fatty food to complete a full course of 6 doses over 3 days, equivalent to a total dosage of 6.2 mg/kg of artemether and 37.4 mg/kg of lumefantrine. Glucose 6-phosphate dehydrogenase function was normal, and a single 30-mg dose of primaquine was administered on day 2. His hemoglobin was 126 g/dL and he received no blood transfusion. After treatment, his parasitemia declined to 1,269/µL at 32 hours, 488/µL at 41 hours, and 55/µL at 56 hours. He was afebrile and symptom-free within 36 hours of admission. However, before discharge on day 6, thick blood film examination was still positive (192/µL), but by day 11, his repeat blood film examination and his aldolase rapid diagnostic test results were negative.

The patient remained in urban Darwin but returned to the hospital 52 days later, on April 22, 2015, with a 2-week history of fevers, fatigue, and headache. Microscopy again identified *P. malariae* with a parasite count of 3,332/µL (Technical Appendix Figure 1, panels C, D). Chloroquine was unavailable, so the patient was re-treated with oral hydroxychloroquine with an 800-mg loading dose, followed by 400 mg at 6 hours, 400 mg at 24 hours, 400 mg at 48 hours, and a single 45-mg dose of oral primaquine. The parasite count declined rapidly to 37/µL at 28 hours, 191/µL at 49 hours, and 76/µL at 88 hours of treatment. His symptoms resolved rapidly. Thick and thin blood films were negative on day 4 and remained negative on retesting at days 8, 35, 41, and 84, and the patient remained free of symptoms throughout. A PCR on blood collected at 12 weeks was also negative.

### Whole-Genome Sequencing

Extensive sequencing was performed from blood samples obtained from the initial (PmUG01) and recrudescent (PmUG02) infection (Technical Appendix Table 1), covering >99% of the genome at >20× for both infections. By using additional *P. malariae* samples published previously (*15*), we identified single-nucleotide polymorphisms (SNPs) using GATK’s UnifiedGenotyper (Broad Institute, Cambridge, MA, USA) (*17*) and filtered them based on several parameters (Technical Appendix Table 2). A multidimensional scaling plot of the samples based on their SNP allele frequency-spectra revealed that PmUG01 and PmUG02 were more closely related to each other than to any of the other samples (Technical Appendix Figure 2), as expected if they were related recombinants derived from the same original infection.

Searching solely for SNPs that distinguish PmUG01 and PmUG02, we identified 2,631 variants after filtering (Technical Appendix Table 2). PmUG01 was the sample from which the reference genome (R1) was constructed (*15*), and only 1 SNP in PmUG01 suggested a nucleotide base different from the reference strain, probably because it was in a repetitive region (Technical Appendix Table 4). PmUG01 appeared to be a polyclonal infection with a bimodal distribution of alternate (i.e., nonreference) alleles at frequencies of 0.15 and 0.35 (Technical Appendix Figure 3, panel A). Conversely, PmUG02 appeared to be a monoclonal infection with ≈85% of sites being either fixed for the reference allele or for an alternative allele (Technical Appendix Figure 3, panel B). Comparison of the initial and recrudescent infections revealed that heterozygous sites in the initial infection had become either homozygous alternate (≈40%) or homozygous reference (≈45%) (Technical Appendix Table 4). Analysis of the genotype calls across the genome (Technical Appendix Figure 4) revealed that, whereas the heterozygous sites from the initial infection were spread evenly across the 14 chromosomes, the homozygous alternate sites in the recrudescent infection were present in distinct clusters, implying that the initial infection was polyclonal and that the recrudescence was attributable to a single clone that was closely related to the reference clone.

**Figure 2:**
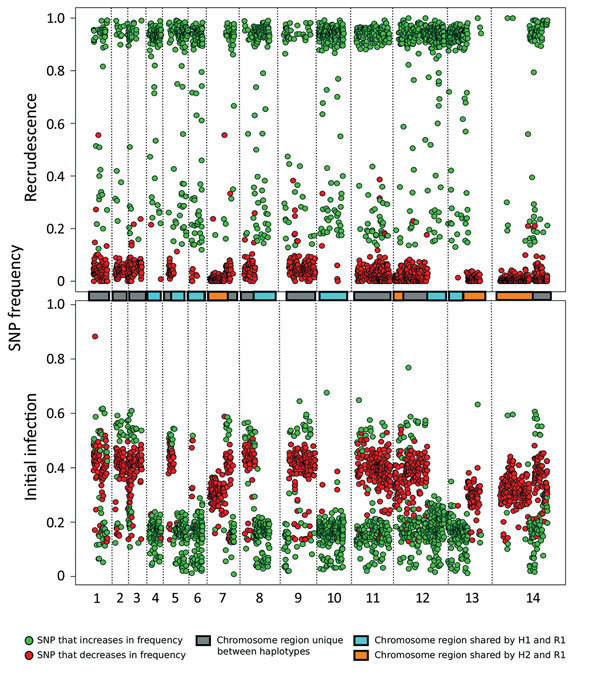
Analysis of the minor haplotype (H2) that caused recrudescence of *Plasmodium malariae* infection in a patient at Royal Darwin Hospital, Darwin, Northern Territory, Australia, March–April 2015, showing distribution of SNP alternative (nonreference) allele frequencies across the 14 chromosomes (boxes in the middle and dotted vertical lines) in the initial infection (bottom plot) and the recrudescence (top plot). The SNP colors (green, increasing in frequency; red, decreasing in frequency) form 2 clear bands, corresponding to H1 (yellow box) and H2 (pink box). H2 probably caused the recrudescence given that all of its alleles increase considerably in frequency. Colored boxes in center of chart indicate chromosome sharing. H1, haplotype 1; H2; haplotype 2; R1, reference genome; SNP, single-nucleotide polymorphism.

Comparison of the distribution of the alternate allele frequencies throughout the genome of the initial and recrudescent strains (Figure 2) revealed bands of alleles at frequencies of ≈0.15 and ≈0.35 in the initial infection spatially clustered throughout the genome. The alleles that increased in relative frequency in PmUG02 were mostly at frequencies of ≈0.15, whereas the alleles at frequencies of ≈0.35 decreased in frequency and the positions became homozygous reference in PmUG02 (Figure 2). These data strongly suggested that, in addition to R1, 2 minor clones (minor haplotypes) were also present. Of these 2, the clone with the haplotype comprising alternate alleles at frequencies of ≈0.35 (H1) appeared to have been eliminated during the drug treatment because no alleles specific to H1 were present in the recrudescent infection. The other minor clone comprised a haplotype with alternate alleles at frequencies of ≈0.15 (H2) in the initial infection; this clone appeared to have caused the recrudescence (Figure 1, panel C). Based on the relative alternate allele frequencies of the 3 haplotypes in the initial infection, ≈60% of the parasites were of the R1 haplotype, 30% of H1, and 10% of H2. These estimates were broadly consistent with the ratio of alleles in tri-allelic sites (0.69:0.22:0.09) (Technical Appendix Table 5). The ratio of alleles in these tri-allelic sites changes markedly in PmUG02 (0.13:0.06:0.81), with over half of sites becoming homozygous for H2 but with some heterogeneity in the other sites (Technical Appendix Table 6), probably because of the low coverage depth and because they were in repetitive regions.

Unexpectedly, several SNPs at high allele frequencies (>0.4) also increased in frequency in the recrudescent strain. Testing by using additional *P. malariae* samples (*15*) showed that ≈80% of these SNPs were homozygous for the alternate allele in >1 other *P. malariae* samples, whereas ≈30% were homozygous in all other *P. malariae* samples (Technical Appendix Figure 5). This observation indicated that of these unusual SNPs, ≈50% were highly polymorphic, whereas ≈30% were probably low-frequency SNPs with rare variants present in the reference strain. This would explain the observation of SNPs with high reference allele frequency in the initial infection that became homozygous alternate in the recrudescence, given that they were probably SNPs with alternate alleles shared by H1 and H2.

To clarify the relationships of the different haplotypes with each other, we classified every genome region by whether any of the 3 haplotypes were identical to each other (Figure 2; Technical Appendix). Approximately 25% of the genome is shared between H1 and R1 and between H2 and R1. No regions were shared between H1 and H2, which suggested that both H1 and H2 were half-siblings of R1, although they did not share any parent between themselves (Technical Appendix Figure 6). The finding that all haplotypes were related to each other through R1 further suggested that all strains were transmitted from the same mosquito bite and that the mosquito ingested at least 4 different parental haplotypes (Technical Appendix Figure 6).

Analysis of SNPs in orthologs of known drug-resistance genes identified 3 nonsynonymous SNPs in the multidrug resistance protein 2 (*mdr2*) gene, 1 of which was in the ABC transporter domain, and 2 in the ABC transporter domain of ABC transporter C family member 2, present in the recrudescent strain (H2) but not the other strains (Technical Appendix Table 7). No evidence was found for copy number variation in any gene compared with the reference strain, and the reference strain did not appear to have an amplification of the multidrug resistance protein 1 gene compared with any of the other *P. malariae* samples.

## Discussion

This report of a case of recurrent *P. malariae* malaria is unusual in that it describes the molecular characterization and confirmation of a treatment failure after directly observed, appropriately administered, quality-assured AL dosing in a nonendemic environment where reinfection was not possible. Whole-genome sequencing demonstrated that the recrudescence was attributable to a minor clone present in the initial polyclonal infection. The case raises 2 important questions: first, what was the cause of treatment failure; and second, why did recrudescence arise from the minor clone rather than a dominant reference clone?

Although the efficacy of AL for *P. malariae* infection is assumed in many national guidelines (*18*), *P. malariae* monoinfections are relatively unusual and often of low density. To our knowledge, there have been no published efficacy series of AL with the long follow-up necessary to assess efficacy against a parasite with a 72-hour life cycle. In a nonrandomized efficacy study of 4 PCR-confirmed *P. malariae* infections treated with AL in Gabon (1 *P. malariae* monoinfection and 3 mixed *P. malariae/P. falciparum* infections), all 4 were microscopy negative at day 28, with no follow-up beyond this time (*19*). Among 80 PCR-confirmed *P. malariae/P. falciparum* mixed species infections in Uganda, 12% were still PCR-positive for *P. malariae* at day 7 and 6% were still PCR-positive on day 17 (*20*). An additional 3 reports have documented *P. malariae* infections occurring at 38 days, 47 days, and 4 months after AL treatment of an initial microscopy-diagnosed *P. falciparum* infection in returned travelers with no further possible malaria exposure (*21*–*23*).

**Figure 3:**
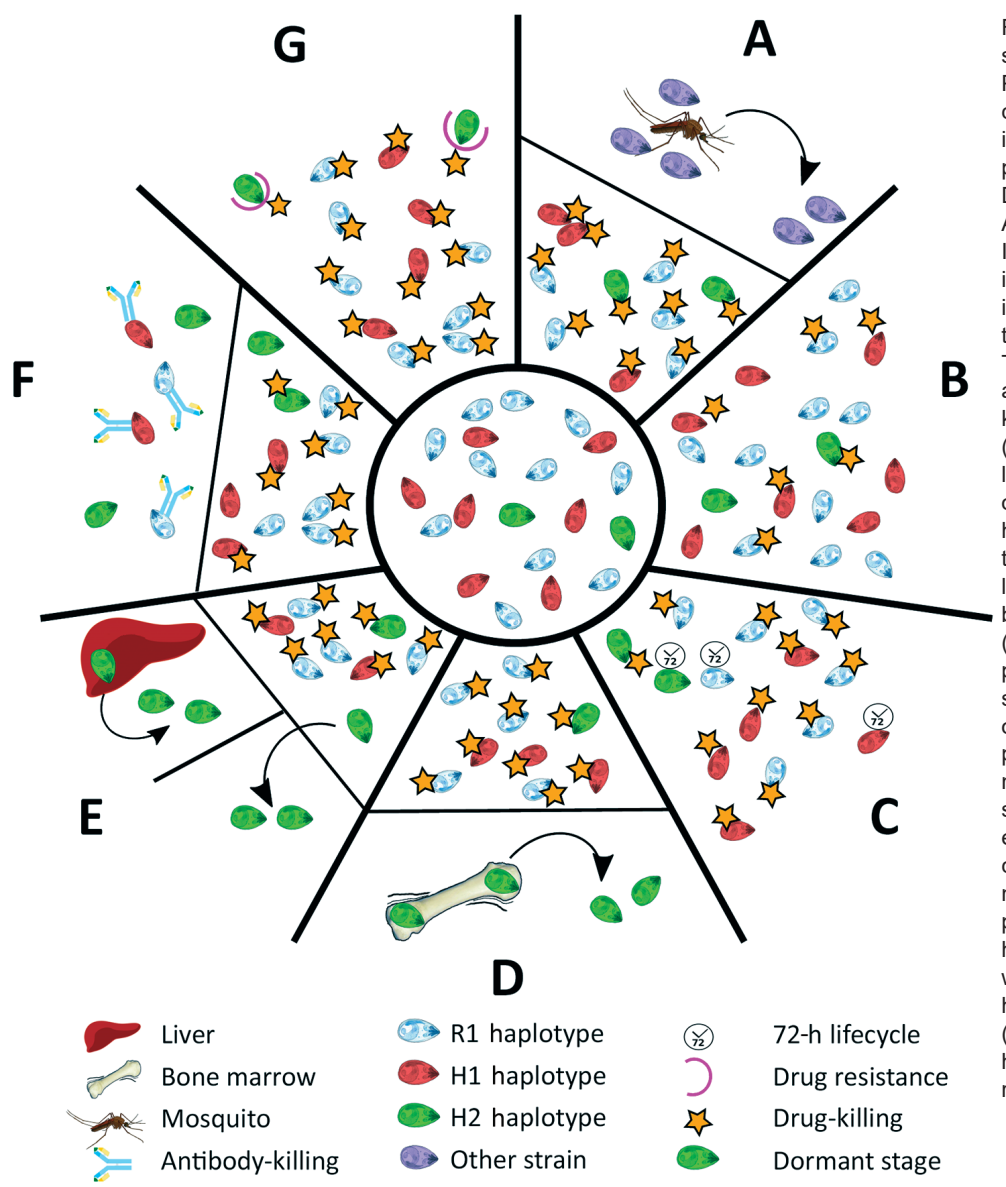
The different scenarios under which a second *Plasmodium malariae* infection could have occurred from the initial infection diagnosed in a patient at Royal Darwin Hospital, Darwin, Northern Territory, Australia, March–April 2015. Initial infection is shown in the inner circle. A) A completely new infection might have caused the second malaria onset. B) The drug might not have been absorbed at sufficient levels to kill all the parasites in the blood (pharmacokinetic cause). C) The longer intraerythrocytic life cycle of *P. malariae* (72 hours) might have enabled some parasites to survive the drug action until lumefantrine concentrations became subtherapeutic (pharmacokinetic cause). D) H2 parasites might have differentially sequestered with a biomass out of proportion with the peripheral parasitemia. E) Some parasites might have formed dormant stages in the liver, blood, or elsewhere (pharmacodynamic cause). F) An immune response might have been differentially primed against haplotypes at higher biomass. G) A haplotype within the initial infection might have been relatively drug resistant (fitness advantage). H1, haplotype 1; H2, haplotype 2; R1, reference genome.

Several plausible explanations might account for a recurrence of *P. malariae* parasitemia after treatment with AL (Figure 3). The last indigenous case of malaria in the Northern Territory was in 1962, with no subsequent cases of introduced malaria or autochthonous transmission (*24*); hence, the possibility of reinfection can be excluded (Figure 3, panel A). Additionally, the presence of the H2 haplotype in the initial infection and recrudescent infection confirms treatment failure.

Inadequate drug absorption resulting in suboptimal serum drug concentrations can cause treatment failure (Figure 3, panel B). Artemether is rapidly absorbed and eliminated (half-life of a few hours), whereas lumefantrine is variably absorbed and more slowly eliminated (half-life ≈3.2 days) (*25*). Lumefantrine is a lipophilic compound with erratic bioavailability unless administered with a small fatty meal (*26*), and for this reason, guidelines recommend administration of AL with a fatty meal such as milk or a small biscuit. In the case of our patient, we were unable to confirm adequate serum concentrations of lumefantrine; however, the patient took a complete course of treatment, and all doses were supervised in the hospital and administered with a milk biscuit to ensure good absorption. None of the treatment doses were vomited. In this scenario, one would expect >98% efficacy against *P. falciparum* (*27*). In addition, the clones associated with the R1 and H1 haplotypes, accounting for ≈90% of the parasite load, were cleared, suggesting that the plasma drug concentrations were sufficient to eliminate both infections. Nevertheless, considerable inter-individual variation exists in lumefantrine exposure, and this patient may have had relatively low concentrations.

Cure of malaria in a nonimmune patient requires that antimalarial blood concentrations are sustained above the parasites’ MIC until the entire parasite biomass has been eliminated. In the presence of antimalarial drugs, the parasite biomass generally decreases over time in an exponential manner, with drug concentrations needing to be sustained above the MIC for >4 life cycles (*28*). In the case of our patient, the baseline parasitemia at initial presentation was 12,000/µL, which is relatively high compared with most *P. malariae* clinical infections (*6*). Thus, the combination of the long parasite life cycle such that 1 rather than 2 asexual cycles were exposed to artemether, and the short period (≈16 days) for which lumefantrine was at concentrations sufficient to kill the parasite may have resulted in parasites surviving the initial treatment and reestablishing a chronic parasitemia that was then sustained for 50 days before recrudescing (Figure 3, panel C).

Another possibility is that some parasites could have sequestered (Figure 3, panel D) or become dormant (Figure 3, panel E). Whereas dormancy would allow a proportion of the parasites to evade blood-stage schizontocidal activity, plausible sites for sequestration of *P. malariae*–infected erythrocytes would still be exposed to therapeutic concentrations of blood-stage antimalarials, making this possibility an unlikely explanation for this patient’s recrudescent infection. *P. malariae* is well-recognized as having a prolonged preerythrocytic phase and a prepatent period of 16–59 days (*6*). The initial treatment course of AL was administered 56 days after the patient left Uganda, so any preerythrocytic stages were probably not present at the time of initial AL treatment.

Although the ability to form hypnozoites (dormant exoerythrocytic stages) occurs in 3 human malaria parasite species (*P. vivax*, *P. ovale curtisi*, and *P. o. wallikeri*), the evidence that latent exoerythrocytic stages do not occur in *P. malariae* is limited (*6*). Case reports have documented *P. malariae* producing symptomatic disease many years after exposure to infection, as noted in the case of a 74-year-old woman in Greece with *P. malariae* reactivation after >40 years (*29*). Such latency suggests that low-level parasitemia could persist for many years after infection, and indeed may be lifelong. In the case of our patient, parasite recrudescence occurred >100 days after he had left a malaria-endemic area.

Although inadequate drug absorption (Figure 3, panel B), duration of treatment (Figure 3, panel C), or dormancy (Figure 3, panel E) all may have contributed to parasite recrudescence, these indiscriminate explanations would be expected to occur primarily in the dominant strain during the initial infection (*28*) (Technical Appendix Figure 7). One could speculate that the H2 minor parasite population might have emerged from a hepatic schizont that ruptured days after those giving rise to the majority haplotypes and, despite genetic similarity, had substantial differences in surface antigenicity. The antibody response to the primary infection, which would have reached a maximum ≈3 weeks after the illness began, would have been directed against the majority haplotypes and might not have recognized the minor population (Figure 3, panel F). Alternatively, more of the minor population might have been in the dormant state compared with the dominant circulating clones with R1 or H1 haplotypes, or more might have been at a higher biomass in erythrocytes sequestered elsewhere, enabling a proportion to evade antimalarial drug action and recrudesce.

Finally, the minority clone with H2 haplotype might have recrudesced because of a fitness advantage over the other clones/haplotypes (Technical Appendix Figure 7, panels B, C), possibly including relative resistance to either artemether or lumefantrine (Figure 3, panel G). In *P. falciparum,* resistance to artemether is acquired through mutations in the propeller domain of *K13* (*30*), whereas *P. falciparum* resistance to lumefantrine is associated with mutations and copy number variation in the *Pfmdr1* gene (*31*,*32*). Although neither of these genes had nonsynonymous mutations in H2, 1 nonsynonymous mutation was noted in the ABC transporter domain of *mdr2*, potentially involved in artemisinin resistance (*33*,*34*), and 2 nonsynonymous mutations were noted in the multidrug resistance–associated protein 2 gene, which has been implicated in reduced ex vivo susceptibility to lumefantrine in *P. falciparum* (*35*). We also identified 2 nonsynonymous SNPs in the dihydrofolate reductase homologue. Low serum concentrations, a modest reduction in lumefantrine efficacy, the prolonged life cycle of *P. malariae*, and rapid elimination of lumefantrine all might have contributed to the observed treatment failure in our patient.

In conclusion, we have described a case of *P. malariae* recrudescence occurring in a non–malaria-endemic country after adequately administered AL. Whole-genome sequencing data revealed that the monoclonal recrudescence consisted of a minor haplotype that accounted for ≈10% of the initial infection and that all the haplotypes in the initial infection were related to each other and therefore probably originated from the same infective mosquito bite. Although the haplotypes were closely related, the genomic data suggest that >4 parental haplotypes were ingested by the mosquito, indicating considerable diversity and transmission of *P. malariae.* This case raises concerns about the adequacy of ACTs with a short half-life partner drug, such as AL, in treating *P. malariae* infections and suggests that optimal ACTs to treat *P. malariae* should include a slowly eliminated partner drug. Our findings reinforce the importance of a longer duration of follow-up monitoring of patients infected with *P. malariae* for late recrudescence.

Technical Appendix Extended methods and additional results for case of a patient with recrudescence *Plasmodium malariae* infection diagnosed and treated at Royal Darwin Hospital, Darwin, Northern Territory, Australia, March–April 2015.
