# Supplementary material for: Genomic Characterization of Recrudescent Plasmodium malariae after Treatment with Artemether/Lumefantrine
Source: Emerg Infect Dis. 2017 Aug;23(8):1300–7. doi: 10.3201/eid2308.161582 (PMC5547787; doi:10.3201/eid2308.161582)
Supplement: Technical Appendix — Extended methods and additional results for case of a patient with recrudescence Plasmodium malariae infection diagnosed and treated at Royal Darwin Hospital, Darwin, Northern Territory, Australia, March–April 2015. [file 16-1582-Techapp-s1.pdf]

Article DOI: https://doi.org/10.3201/eid2308.161582

Genomic Characterization of Recrudescent *Plasmodium malariae* after Treatment with Artemether/Lumefantrine

Technical Appendix

Supplementary Methods

Ethics Statement

The protocol used to collect human blood samples for patients with malaria attending Royal Darwin Hospital was approved by the Health Research Ethics Committee of Menzies School of Health Research (HREC 09/83). Written informed consent was obtained from the patient.

Sample Collection

Plasmodium malariae DNA used in this study was isolated from a symptomatic patient who presented to the Royal Darwin Hospital, Australia in March and April 2015 with a *P. malariae* parasitemia detected by blood film examination. At each episode, 5ml of EDTA blood was collected from the patient for routine confirmation of malaria by microscopy, full blood count, urea and electrolytes and liver function tests. An additional 10ml of EDTA blood was collected and leukodepleted by passage through a Plasmodipur filter (Euro-diagnostica) within 6 hours of collection. DNA was extracted from a 2ml aliquot of the filtered red blood cell pellet using the QIAamp DNA Blood Midi Kit (Qiagen) as per the manufacturer's instructions, and stored at −20°C. *Plasmodium* species was confirmed by PCR for *P. vivax, P. falciparum*, *P. malariae* and *P. ovale* parasites using a modified version of that described by Padley et al. (*1*) so that each species was identified in a separate (non-multiplex) assay. PCR for *P. knowlesi* parasites was undertaken using the method of Imwong et al. (*2*).

Genome Sequencing

Whole genome sequencing was performed on both parasite isolates (PmUG01 and PmUG02) using Illumina Standard libraries of 200–300bp fragments and amplification-free

libraries of 400–600bp fragments were prepared (*3*) and sequenced on the Illumina HiSeq 2000 v4, the MiSeq v2, and the X Ten according to the manufacturer's standard protocol. Raw sequence data were deposited in the European Nucleotide Archive (Technical Appendix Table 1).

Genotyping of Single Nucleotide Variants (SNPs)

The two *P. malariae* samples (PmUG01, PmUG02) were mapped against the *P. malariae* reference genome (*4*) using SMALT (-y 0.8, -i 500). The resulting bam files were merged, and GATK's (*5*) UnifiedGenotyper was used to call SNPs from the merged bam files (Technical Appendix Table 2). According to GATK's (*5*) best practices, SNPs were filtered by quality of depth (QD >2), depth of coverage (DP >20), mapping quality (MQ >30), and strand bias (FS <60). SNPs in low-complexity regions, as determined by Dustmasker (*6*), were removed, as were sites with missing data in either of the two samples, and SNPs within 50bp of each other to avoid SNPs in repetitive regions. Finally, only exonic SNPs were retained. We performed the same SNP calling procedure by also including additional previously published samples (*4*). Heterozygous sites were filtered out, while SNPs in non-coding regions were retained (Technical Appendix Table 3). Raw SNPs for PmUG01 and PmUG02 differ between Technical Appendix Tables 2 and 3 due to calling SNPs from a merged bam file instead of individually. Samples are therefore pooled and SNP calling is performed on the population rather than on the individual. The consequence of this is that if an individual sample has insufficient reads at a particular locus to reliably call a SNP there, that SNP might still be called if other samples in the population also have SNPs at that location, as this increases the likelihood of a SNP at that position. The number of SNPs therefore differs for identical samples depending on the population on which the SNP calling was performed.

Abundance Calculations

Using the relative SNP frequencies of the three haplotypes (R1, H1 and H2), the relative abundances of the different haplotypes were calculated, assuming that the number of sequencing reads is proportional to the abundance of the specific haplotype in the blood. From the SNP frequencies, H1 and R1 are in a ratio of 0.35:0.65 and H2 to R1 is in a ratio of 0.15:0.85. In both cases we ignore the third haplotype because we cannot ascertain its genotype. Ratio multiplication yields a joint ratio of 975:2975:5525 for H2:H1:R1, simplifying to ≈10:30:60. Tri-allelic sites offer the most straightforward way of observing the ratio of the three haplotypes,

however their number is low. Following SNP filtering (see above), the retained 13 tri-allelic sites were spread evenly across the genome (Technical Appendix Table 5). Assuming that the allele with the highest depth is R1, the intermediate depth is H1, and lowest depth is H2, we calculated the mean depth for all three. This yielded a ratio of ≈9:22:69 for H2:H1:R1 (Technical Appendix Table 5).

References

1. Padley D, Moody AH, Chiodini PL, Saldanha J. Use of a rapid, single-round, multiplex PCR to detect malarial parasites and identify the species present. Ann Trop Med Parasitol. 2003;97:131–7. PubMed http://dx.doi.org/10.1179/000349803125002977

2. Imwong M, Tanomsing N, Pukrittayakamee S, Day NP, White NJ, Snounou G. Spurious amplification of a *Plasmodium vivax* small-subunit RNA gene by use of primers currently used to detect *P. knowlesi*. J Clin Microbiol. 2009;47:4173–5. PubMed http://dx.doi.org/10.1128/JCM.00811-09

3. Bronner IF, Quail MA, Turner DJ, Swerdlow H. Improved protocols for illumina sequencing. Curr Protoc Hum Genet. 2014;80:18.2.1–42. PubMed https://doi.org/10.1002/0471142905.hg1802s80

4. Rutledge GG, Böhme U, Sanders M, Reid AJ, Cotton JA, Maiga-Ascofare O, et al. *Plasmodium malariae* and *P. ovale* genomes provide insights into malaria parasite evolution. Nature. 2017;542:101–4. PubMed http://dx.doi.org/10.1038/nature21038

5. McKenna A, Hanna M, Banks E, Sivachenko A, Cibulskis K, Kernytsky A, et al. The Genome Analysis Toolkit: a MapReduce framework for analyzing next-generation DNA sequencing data. Genome Res. 2010;20:1297–303. PubMed http://dx.doi.org/10.1101/gr.107524.110

6. Morgulis A, Gertz EM, Schäffer AA, Agarwala R. A fast and symmetric DUST implementation to mask low-complexity DNA sequences. J Comput Biol. 2006;13:1028–40. PubMed http://dx.doi.org/10.1089/cmb.2006.13.1028

7. Rutherford K, Parkhill J, Crook J, Horsnell T, Rice P, Rajandream MA, et al. Artemis: sequence visualization and annotation. Bioinformatics. 2000;16:944–5. PubMed http://dx.doi.org/10.1093/bioinformatics/16.10.944

8. Fidock DA, Nomura T, Talley AK, Cooper RA, Dzekunov SM, Ferdig MT, et al. Mutations in the *P. falciparum* digestive vacuole transmembrane protein PfCRT and evidence for their role in chloroquine resistance. Mol Cell. 2000;6:861–71. PubMed http://dx.doi.org/10.1016/S1097-2765(05)00077-8

9. MalariaGEN *Plasmodium falciparum* Community Project. Genomic epidemiology of artemisinin resistant malaria. Elife. 2016;5:5. PubMed https://doi.org/10.7554/eLife.08714

10. Henriques G, Martinelli A, Rodrigues L, Modrzynska K, Fawcett R, Houston DR, et al. Artemisinin resistance in rodent malaria—mutation in the AP2 adaptor μ-chain suggests involvement of endocytosis and membrane protein trafficking. Malar J. 2013;12:118. PubMed http://dx.doi.org/10.1186/1475-2875-12-118

11. Henriques G, Hallett RL, Beshir KB, Gadalla NB, Johnson RE, Burrow R, et al. Directional selection at the pfmdr1, pfcrt, pfubp1, and pfap2mu loci of *Plasmodium falciparum* in Kenyan children treated with ACT. J Infect Dis. 2014;210:2001–8. PubMed http://dx.doi.org/10.1093/infdis/jiu358

12. Dahlström S, Ferreira PE, Veiga MI, Sedighi N, Wiklund L, Mårtensson A, et al. *Plasmodium falciparum* multidrug resistance protein 1 and artemisinin-based combination therapy in Africa. J Infect Dis. 2009;200:1456–64. PubMed http://dx.doi.org/10.1086/606009

13. Dahlström S, Veiga MI, Mårtensson A, Björkman A, Gil JP. Polymorphism in PfMRP1 (*Plasmodium falciparum* multidrug resistance protein 1) amino acid 1466 associated with resistance to sulfadoxine-pyrimethamine treatment. Antimicrob Agents Chemother. 2009;53:2553–6. PubMed http://dx.doi.org/10.1128/AAC.00091-09

14. Veiga MI, Osório NS, Ferreira PE, Franzén O, Dahlstrom S, Lum JK, et al. Complex polymorphisms in the *Plasmodium falciparum* multidrug resistance protein 2 gene and its contribution to antimalarial response. Antimicrob Agents Chemother. 2014;58:7390–7. PubMed http://dx.doi.org/10.1128/AAC.03337-14

15. Okombo J, Abdi AI, Kiara SM, Mwai L, Pole L, Sutherland CJ, et al. Repeat polymorphisms in the low-complexity regions of *Plasmodium falciparum* ABC transporters and associations with in vitro antimalarial responses. Antimicrob Agents Chemother. 2013;57:6196–204. PubMed http://dx.doi.org/10.1128/AAC.01465-13

16. Jambou R, Legrand E, Niang M, Khim N, Lim P, Volney B, et al. Resistance of *Plasmodium falciparum* field isolates to in-vitro artemether and point mutations of the SERCA-type PfATPase6. Lancet. 2005;366:1960–3. PubMed http://dx.doi.org/10.1016/S0140-6736(05)67787-2

17. Spillman NJ, Allen RJ, McNamara CW, Yeung BK, Winzeler EA, Diagana TT, et al. Na(+) regulation in the malaria parasite *Plasmodium falciparum* involves the cation ATPase PfATP4

and is a target of the spiroindolone antimalarials. Cell Host Microbe. 2013;13:227–37. PubMed http://dx.doi.org/10.1016/j.chom.2012.12.006

18. Vaidya AB, Morrisey JM, Zhang Z, Das S, Daly TM, Otto TD, et al. Pyrazoleamide compounds are potent antimalarials that target Na+ homeostasis in intraerythrocytic *Plasmodium falciparum*. Nat Commun. 2014;5:5521. PubMed http://dx.doi.org/10.1038/ncomms6521

19. Jiménez-Díaz MB, Ebert D, Salinas Y, Pradhan A, Lehane AM, Myrand-Lapierre ME, et al. (+)-SJ733, a clinical candidate for malaria that acts through ATP4 to induce rapid host-mediated clearance of *Plasmodium*. Proc Natl Acad Sci U S A. 2014;111:E5455–62. PubMed http://dx.doi.org/10.1073/pnas.1414221111

20. Ariey F, Witkowski B, Amaratunga C, Beghain J, Langlois AC, Khim N, et al. A molecular marker of artemisinin-resistant *Plasmodium falciparum* malaria. Nature. 2014;505:50–5. PubMed http://dx.doi.org/10.1038/nature12876

21. Henry M, Briolant S, Zettor A, Pelleau S, Baragatti M, Baret E, et al. *Plasmodium falciparum* Na+/H+ exchanger 1 transporter is involved in reduced susceptibility to quinine. Antimicrob Agents Chemother. 2009;53:1926–30. PubMed http://dx.doi.org/10.1128/AAC.01243-08

22. Cowman AF, Morry MJ, Biggs BA, Cross GA, Foote SJ. Amino acid changes linked to pyrimethamine resistance in the dihydrofolate reductase-thymidylate synthase gene of *Plasmodium falciparum*. Proc Natl Acad Sci U S A. 1988;85:9109–13. PubMed http://dx.doi.org/10.1073/pnas.85.23.9109

23. Brooks DR, Wang P, Read M, Watkins WM, Sims PF, Hyde JE. Sequence variation of the hydroxymethyldihydropterin pyrophosphokinase: dihydropteroate synthase gene in lines of the human malaria parasite, *Plasmodium falciparum*, with differing resistance to sulfadoxine. Eur J Biochem. 1994;224:397–405. PubMed http://dx.doi.org/10.1111/j.1432-1033.1994.00397.x

24. Srivastava IK, Morrisey JM, Darrouzet E, Daldal F, Vaidya AB. Resistance mutations reveal the atovaquone-binding domain of cytochrome b in malaria parasites. Mol Microbiol. 1999;33:704–11. PubMed http://dx.doi.org/10.1046/j.1365-2958.1999.01515.x

Technical Appendix Table 1. Sequencing Information and Statistics

Characteristic	PmUG01	PmUG02
Accession Number	ERS1110316	ERS1110319
Origin	Uganda	Uganda
Infection	Initial	Recrudescence
Mean Coverage Depth	407x	136x
Coverage Range (Min-Max)	0x-7499x	0x-3519x
% Genome Covered at 1x	99.9%	99.9%
Sequencing Platform	Illumina HiSeq X Ten Illumina MiSeq v2 Illumina HiSeq 2000 v4	Illumina HiSeq X Ten Illumina MiSeq v2 Illumina HiSeq 2000 v4
Library Type	Amplification Free	Amplification Free

Technical Appendix Table 2. SNP Calling Results specifically for both PmUG01 and PmUG02

Sample ID	PmUG01	PmUG02
Raw SNPs	274,494	287,610
Private	79,953	93,069
Ref	362,848	333,615
Missing*	298	1,470
Filtered SNPs	2,442	1,499
Private	1,132	189
Ref	189	1,773
Missing*	0	0

*Sites at which the sample has no coverage. SNP calling results as per mapping the two *P. malariae* samples from the present clinical case against the PmUG01 reference genome (*4*). The raw SNPs are the total number of SNPs that we called using GATK's UnifiedGenotyper default parameters in the different samples. Of these raw SNPs, some are exclusive to a certain sample (Private), are identical to the reference genome (Ref), or there is no coverage and therefore no SNP call could be made (Missing). The same information is also shown for the filtered SNPs, which were filtered according to several different parameters (Methods).

Technical Appendix Table 3. SNP Calling Results for all *P. malariae* samples

Sample ID	PmUG01	PmUG02	PmMY01	PmID01	PmMA01	PmGN01
Origin	Uganda	Uganda	Malaysia	Papua Indonesia	Mali	Guinea
Raw SNPs	200,679	191,766	252,172	187,327	198,029	503,175
Private	13,300	10,462	48,632	19,396	26,637	73,208
Ref	531,180	479,142	367,672	347,257	360,155	398,393
Missing*	5,468	23,064	65,361	107,355	95,973	38,609
Filtered SNPs	1,375	2,707	22,696	17,564	16,057	21,329
Private	0	414	8,816	5,939	6,079	10,343
Ref	49,647	47,868	27,521	32,762	34,205	29,021
Missing*	0	0	0	0	0	0

*Sites at which the sample has no coverage. SNP calling results as per mapping all *P. malariae* samples against the PmUG01 reference genome (*4*). The raw SNPs are the total number of SNPs that we called using GATK's UnifiedGenotyper default parameters in the different samples. Of these raw SNPs, some are exclusive to a certain sample (Private), are identical to the reference genome (Ref), or there is no coverage and therefore no SNP call could be made (Missing). The same information is also shown for the filtered SNPs, which were filtered according to several different parameters (Methods).

Technical Appendix Table 4. Changes in genotype calls between the two infections

Initial Genotype	Recrudescence Genotype	Number of Sites
HR	HA	1
h	h	217
h	HR	1,178
h	HA	1,038
HA	h	1*
HR	h	196
HA	HR	0

HR = Homozygous reference HA = Homozygous alternate h = heterozygous
* in repetitive region of rhoptry-associated membrane antigen

Technical Appendix Table 5. Sequencing reads for the three haplotypes in tri-allelic sites in PmUG01

Genomic Location	H2 haplotype reads	H1 haplotype reads	R1 haplotype reads
Chr1: 704,003	6	21	202
Chr2: 437,291	43	71	130
Chr7: 1,580,127	12	18	75
Chr9: 1,095,695	39	91	117
Chr9: 1,429,882	21	82	140
Chr10: 873,165	9	31	209
Chr11: 987,107	17	60	173
Chr11: 2,535,419	47	84	113
Chr12: 2,746,074	9	44	192
Chr12: 2,855,999	11	36	176
Chr12: 3,047,435	11	34	204
Chr13: 1,061,473	13	16	221
Chr14: 1,118,313	27	62	139
Total (Proportion)	265 (0.09)	650 (0.22)	2,091 (0.69)

Assuming that the lower-level genotype is H2, intermediate-level genotype is H1, and higher-level genotype is R1, the tri-allelic sites have a certain number of sequencing reads confirming each of these three genotypes.

Technical Appendix Table 6. Sequencing reads for the three haplotypes in tri-allelic sites in PmUG02

Genomic Location	H2 haplotype reads	H1 haplotype reads	R1 haplotype reads
Chr1: 704,003	16	0	7
Chr2: 437,291	20	4	0
Chr7: 1,580,127	18	0	9
Chr9: 1,095,695	25	0	0
Chr9: 1,429,882	23	0	0
Chr10: 873,165	19	8	1
Chr11: 987,107	11	0	7
Chr11: 2,535,419	15	0	1
Chr12: 2,746,074	9	0	1
Chr12: 2,855,999	6	4	4
Chr12: 3,047,435	16	0	0
Chr13: 1,061,473	8	0	5
Chr14: 1,118,313	26	0	0
Total (Proportion)	212 (0.81)	16 (0.06)	35 (0.13)

Technical Appendix Table 7. H2 specific nonsynonymous and synonymous mutations in drug resistance genes

Gene ID (PmUG01_*)	Gene Description	*P. falciparum* ortholog	nsSNPs	sSNPs	Resistance
01020700	Chloroquine resistance transporter	0709000	0	0	Chloroquine (*8*), Quinine
10021600	Multidrug resistance protein 1	0523000	0	2	Multiple
12069100	Multidrug resistance protein 2	1447900	3	0	Multiple, Artemisinin (*9*)
14053100	AP-3 complex subunit mu	1218300	0	0	Artemisinin (*10*,*11*)
02019300	Ubiquitin carboxyl- terminal hydrolase 1	0104300	0	0	Artemisinin (*11*)
02011900	ABC transporter C family member 1	0112200	0	0	Artemisinin (*12*), Sulfadoxine/Pyrimeth amine (*13*)
14063400	ABC transporter C family member 2	1229100	2	0	Chloroquine (*14*), Piperaquine (*14*), Mefloquine (*14*) Lumefantrine (*15*)
02017400	Calcium-transporting ATPase	0106300	0	0	Artemisinin (*16*)
13021900	P-type ATPase4	1211900	0	1	Spiroindolones (*17*), Pyrazoleamides (*18*), Dihydroisoquinolone s (*19*)
12021200	Kelch protein k13	1343700	0	0	Artemisinin (*20*)
14020100	Sodium/hydrogen exchanger 1	1303500	0	0	Quinine (*21*)
05034700	Bifunctional dihydrofolate reductase- thymidylate	0417200	2	1	Pyrimethamine (*22*)
14036800	ATP-dependent Clp protease adaptor protein	0810800	0	0	Sulfadoxine (*23*)
MIT001100	Cytochrome b	mal_mito_3	0	0	Atovaquone (*24*)

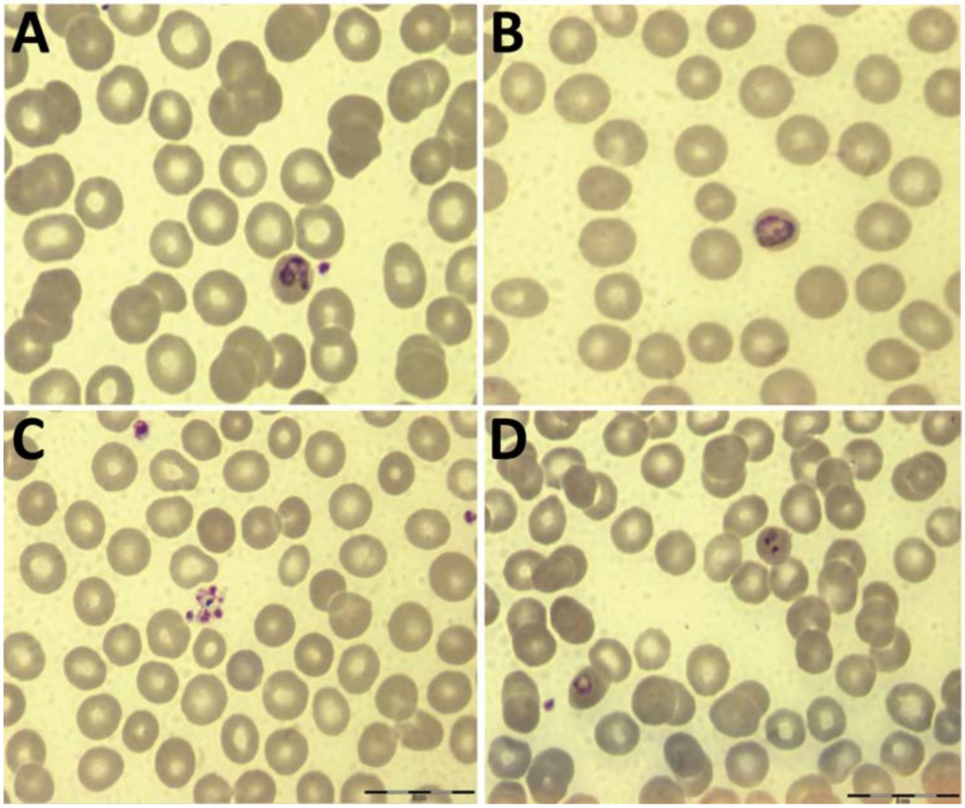

Technical Appendix Figure 1. Positive *Plasmodium malariae* thin blood films. Thin smear scans of the initial infection (A, B) and of the recrudescence (C, D), both indicating a *P. malariae* infection.

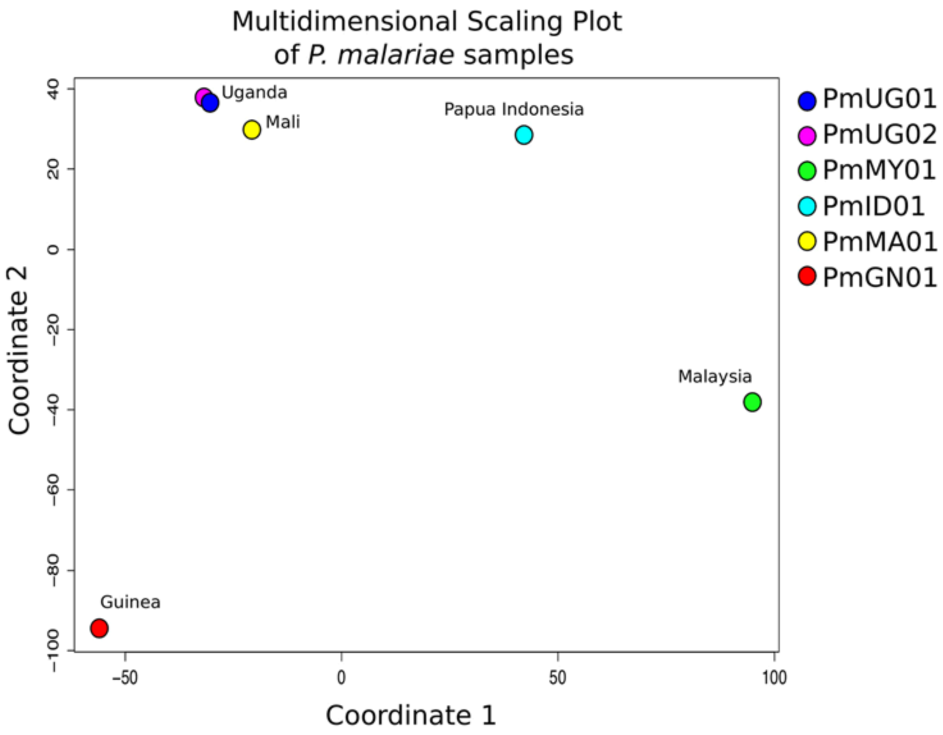

Technical Appendix Figure 2. Similar SNP spectra for PmUG01 and PmUG02. Multidimensional scaling plot based on differences in SNP spectra between the different *P. malariae* samples (*4*), showing that the initial (PmUG01) and the recrudescent (PmUG02) infections are significantly more similar to each other than to the other *P. malariae* samples. This suggests that the two infections have a similar origin.

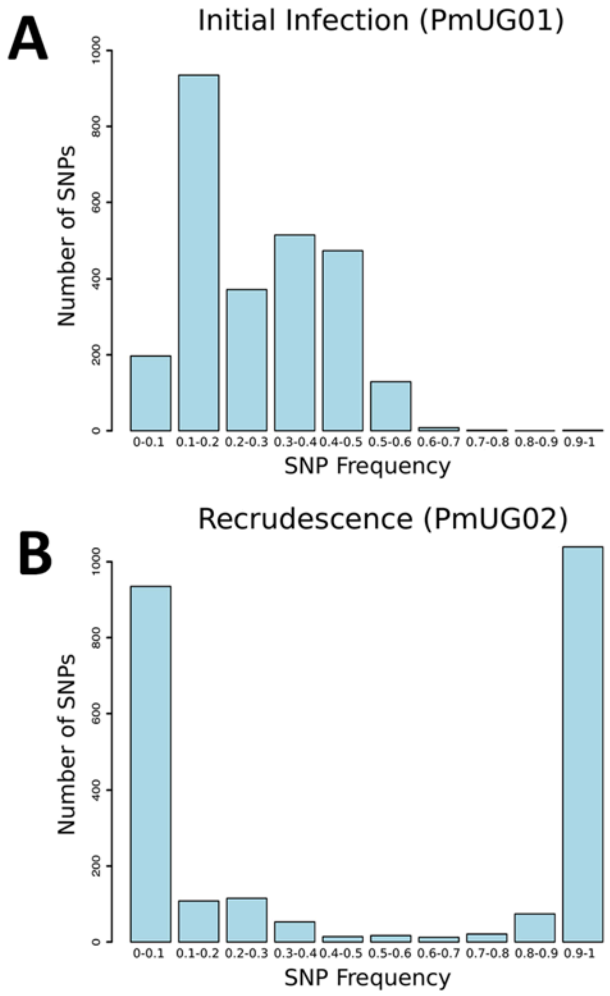

Technical Appendix Figure 3. Differences in SNP frequencies between PmUG01 and PmUG02. SNP frequency bar plots for both the initial infection (A) and the recrudescence (B), showing that there was a significant shift in the SNP frequency spectra between the two infections, with the initial infection being a polyclonal infection, while the recrudescence seems to be monoclonal. Interestingly, the initial infection seems to have a bimodal distribution of heterozygous SNPs.

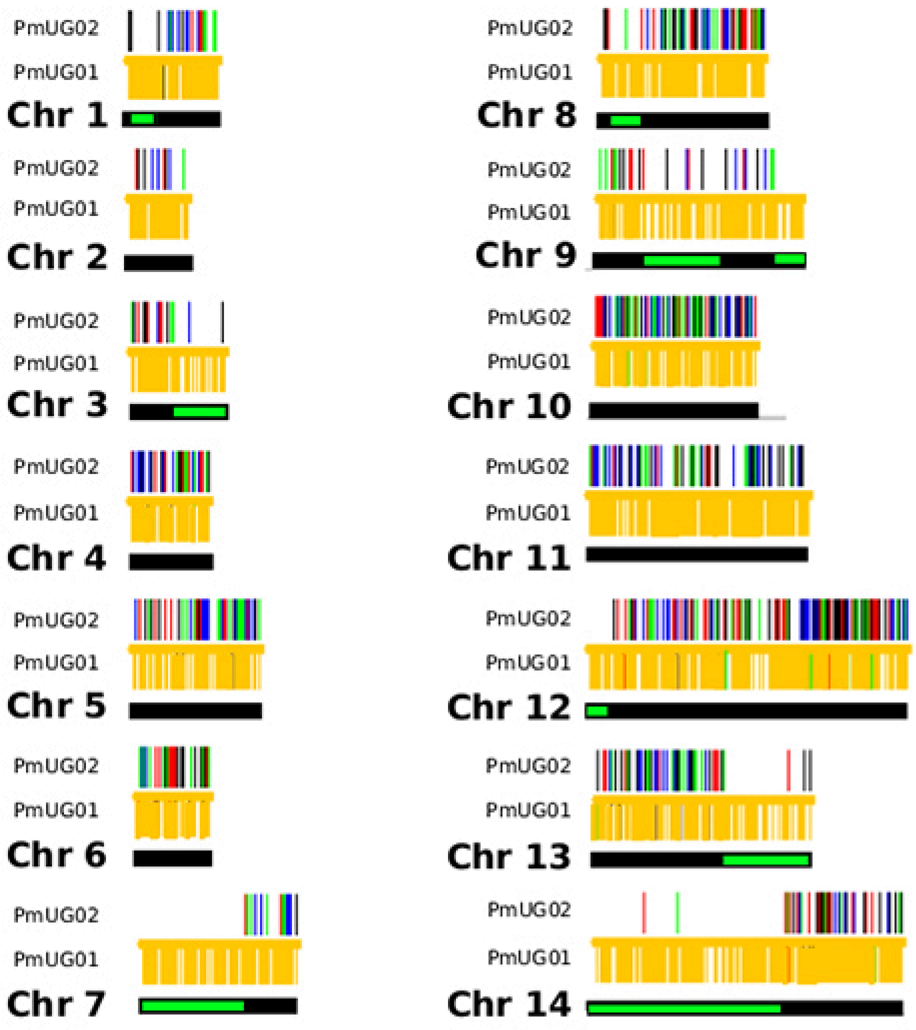

Technical Appendix Figure 4. Clustered SNP distribution in PmUG02 across chromosomes. Distribution of heterozygous sites (yellow) in the initial infection (PmUG01) and homozygous alternate sites (other colors) in the recrudescence (PmUG02) across the 14 chromosomes of *P. malariae*. The different colors for the homozygous alternate SNPs are arbitrary. Chromosome regions with more heterozygous sites in the initial infection becoming homozygous reference than becoming homozygous alternate in the recrudescence are marked in green. Genotypes were plotted using Artemis (*7*).

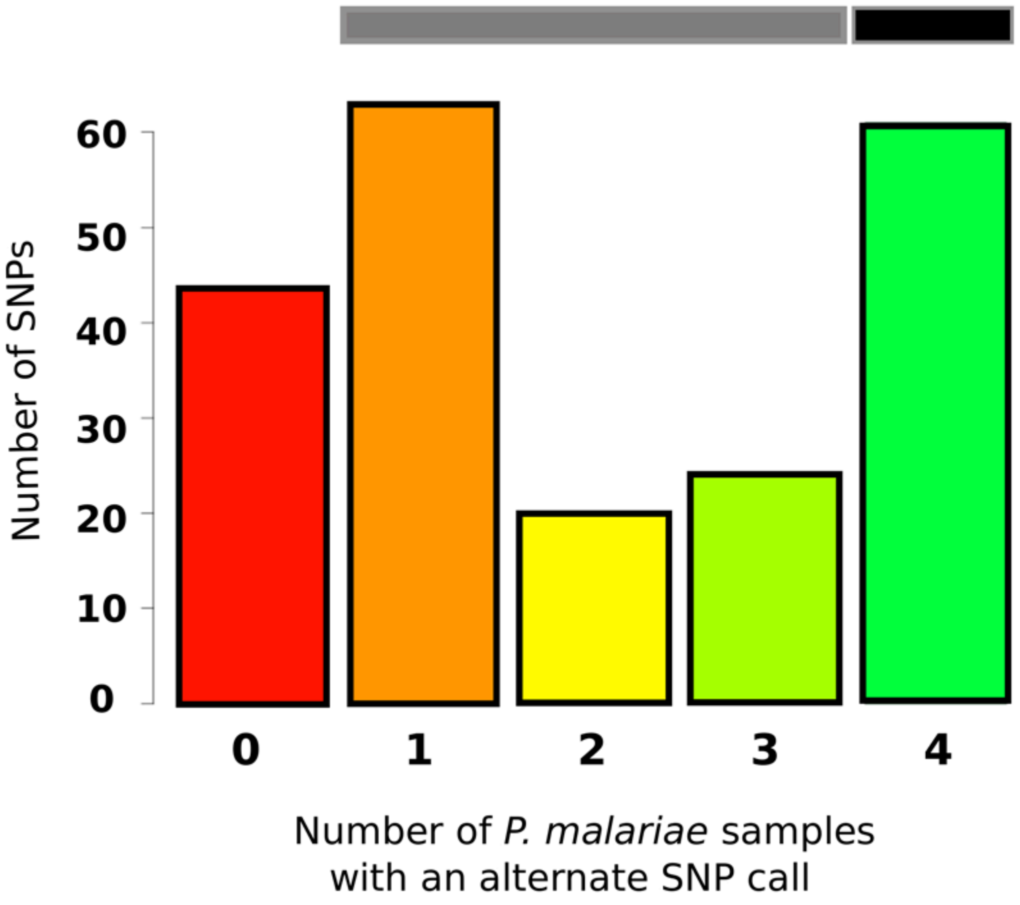

Technical Appendix Figure 5. Unusual SNPs in other *P. malariae* samples. Presence/absence in other *P. malariae* samples of SNPs with SNP frequencies of over 0.4 in the initial infection that increased in frequency in the recrudescence. The black bar indicates SNPs that are present in all other *P. malariae*, suggesting that the reference strain is rather the variant compared to the general population. The gray bar indicates SNPs that are present in multiple other *P. malariae* samples, suggesting that they are highly polymorphic sites. In sum, this suggests that most of these SNPs are likely SNPs shared by both H1 and H2, explaining why they have high frequencies in the initial infection and in the recrudescence.

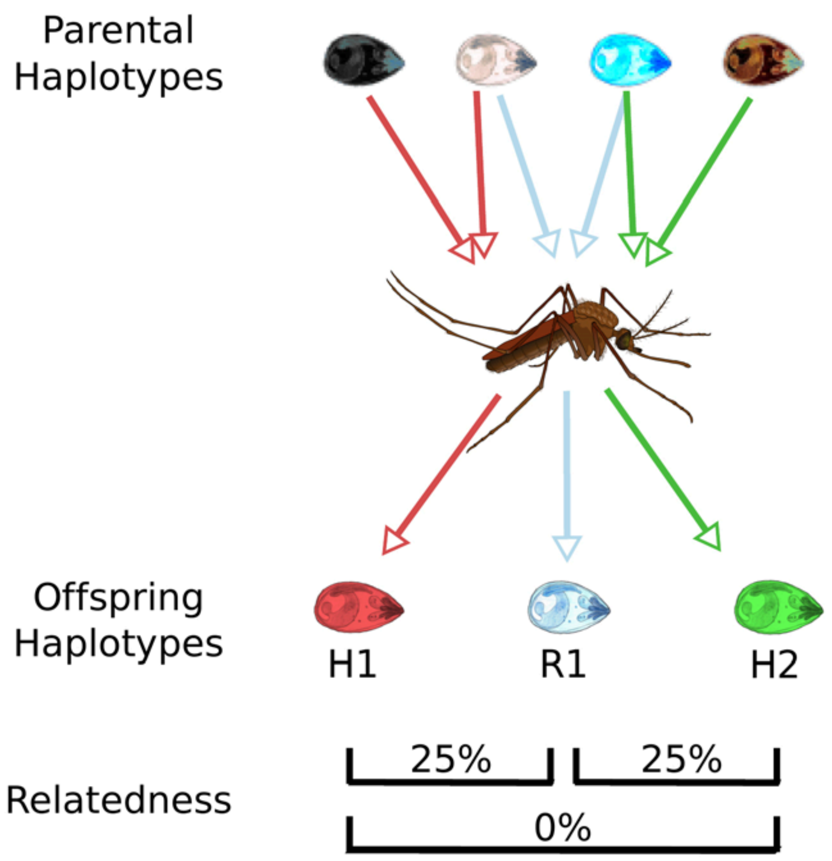

Technical Appendix Figure 6. Inferred number of parental haplotypes. The relatedness of the three haplotypes in the initial infection (offspring haplotypes) as inferred by the sharing of genomic regions. This sharing suggests that there were four parental haplotypes present in the mosquito that interbred to form the three haplotypes we see in the initial infection. Of these, it seems that R1 is a half-sibling with both H1 and H2, but via a different parental haplotype.

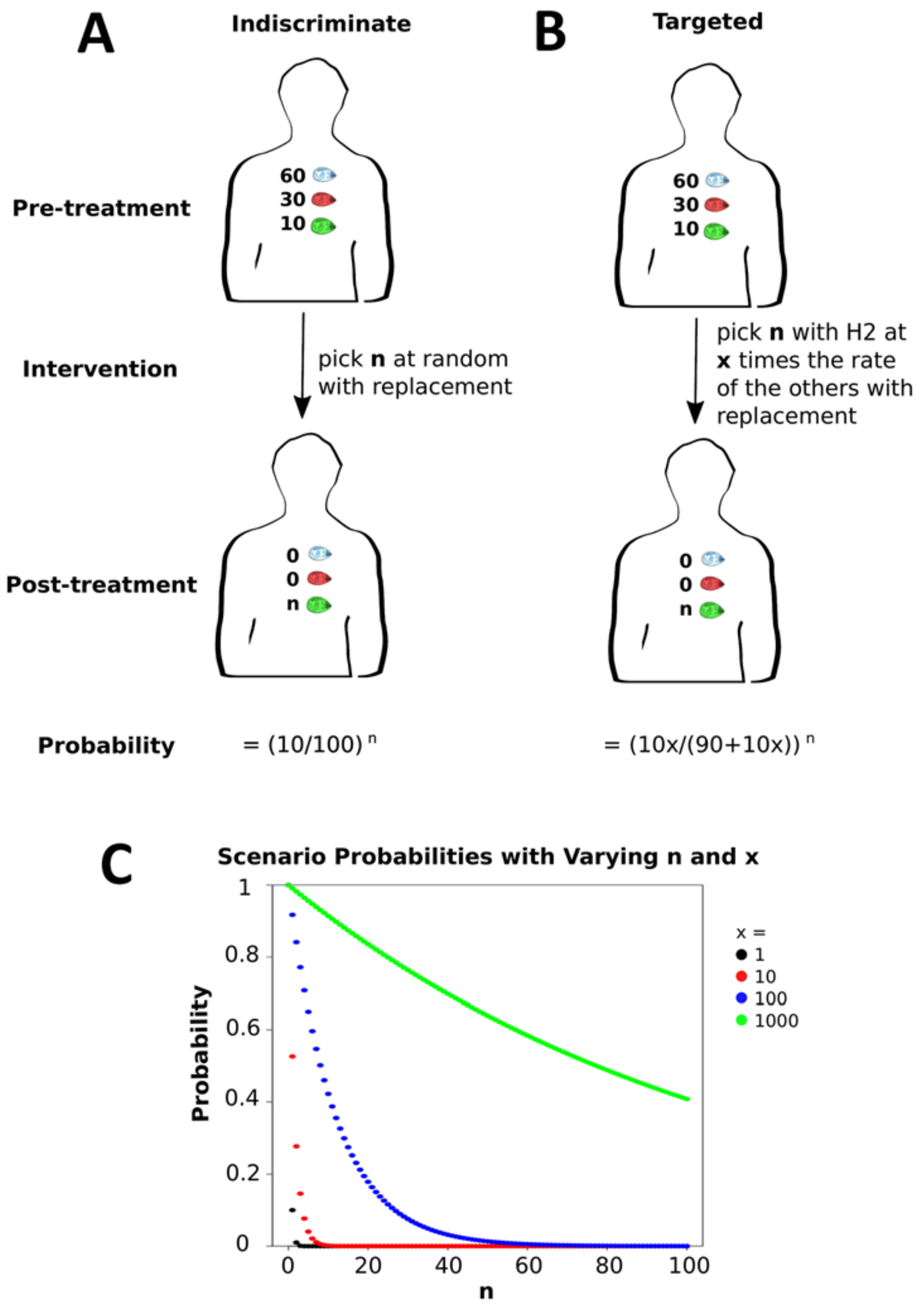

Technical Appendix Figure 7. Indiscriminate versus targeted interventions. Differences in probabilities for (A) indiscriminate interventions (i.e., explanations for a recrudescence) that affect all haplotypes in the infection equally, such as insufficient drug dosage or drug avoidance through a longer lifecycle, versus (B) targeted interventions that potentially affect one haplotype different to another, for example haplotype-specific drug resistance or an increased propensity for greater sequestered biomass in one haplotype. The two scenarios show that the difference is in how an intervention 'selects' for n number of parasites in the recrudescence. In an indiscriminate intervention, all haplotypes have the same probability of being

selected, while H2 is x times more likely to be selected in a targeted intervention. An indiscriminate intervention has a low probability (<0.05) at all values of n > 1 (C), while the probability of a targeted intervention increases across all values of n the higher x. If an indiscriminate intervention were to be the sole explanation for a recrudescence, then it would suggest that only a singly parasite survived from the entire initial infection, an unlikely scenario. On the other hand, a targeted intervention presents a more parsimonious explanation for the lower level haplotype recrudescing.
